# Blood cancer caregivers’ communication with clinicians about online cancer information

**DOI:** 10.1017/S1478951526101692

**Published:** 2026-02-12

**Authors:** Alyssa Crowe, Taylor T. Vasquez, Kevin B. Wright, Carla L. Fisher, Skyler B. Johnson, Samantha R. Paige, Maria Sae-Hau, Elisa S. Weiss, Carma L. Bylund

**Affiliations:** 1 Health Outcomes and Biomedical Informatics, University of Florida, Gainesville, FL, USA; 2 College of Journalism and Communications, University of Florida, Gainesville, FL, USA; 3 Department of Communication, George Mason University, Fairfax, VA, USA; 4 Radiation Oncology, University of Utah Huntsman Cancer Institute, Salt Lake City, USA; 5 SRPW Consulting, LLC Potsdam, NY; 6 Department of Education, Services & Outcomes Research, Blood Cancer United, Inc., Washington D.C., USA

**Keywords:** Caregivers, online health information, physician–patient communciation, health literacy, cancer

## Abstract

**Objectives:**

Adult children caring for a parent with cancer often assume the role of a “surrogate seeker,” looking online for information regarding their parent’s diagnosis, which they may then discuss with their parent’s clinician. The current study aims to apply the previously developed “Stoplight typology” to explore caregivers’ experiences discussing online health information with their parents’ clinicians and factors associated with each response type within the typology.

**Methods:**

We conducted an online survey of adult children caring for a parent with a blood cancer about their experiences communicating with their parent and their parent’s clinicians. We used regression analyses to examine the association between physician responses as categorized according to the stoplight typology with 3 caregiver communication measures assessing eHealth literacy, caregiver communication, and physician–caregiver and patient communication. Second, we examined the experiences participants had with clinician communication about online health information.

**Results:**

A total of 121 caregivers completed the survey. Over half reported clinicians giving green light responses, with fewer reporting yellow or red light responses. Lower eHealth literacy significantly predicted greater likelihood of red light responses, whereas higher self-reported communication skills predicted more green light and fewer red light responses; neither communication measure predicted yellow responses. Thirty-two percent did not discuss their most recent online search with clinicians, most commonly because they saw no need. Seventy-four percent had discussed online information with a clinician, and 56% of these encounters were coded as green light responses. Among caregivers who had been told not to search online, 77% continued to do so despite the clinician’s discouragement.

**Conclusions:**

The study findings provide support for the stoplight typology in a caregiver population. Although most clinician responses were engagement responses, results demonstrate that the rejection response is ineffective. Future research could examine caregivers who reported lower eHealth literacy to target for future intervention.

## Introduction

Family members caring for an individual with cancer often take on several different types of roles including, nurse, nutritionist, and therapist (Katarzyna Woźniak 2014). Adult children in particular are known to take on these roles in addition to balancing other career, family, and spousal obligations (Given et al., 2001; Yaacov G Bachner and Raveis 2009). Additionally, caregivers commonly assume the role of a “surrogate seeker” (Reifegerste et al., 2019; Vasquez et al., 2022a) meaning they seek cancer information online on the patient’s behalf (Cutrona et al., 2015). Surrogate seekers often bring information they read online into the clinical encounter (Bylund et al., 2009; Vasquez et al., 2022a), yet little is documented about how surrogate seekers interact with clinicians when discussing online cancer information and factors that influence these conversations.

Based on observational data collected in prior studies (Bylund et al., 2009, 2010), we previously developed a stoplight typology to describe clinician guidance about online information seeking during the clinical interaction This typology categorizes the different conversational responses used by clinicians when discussing online health information with patients and caregivers into 3 types: *red light* responses, e.g., “you should not search online for health information”); *yellow light* responses (e.g., “you should be cautious with information found online”); and *green light* responses (e.g., “Let’s talk about the information you found” or “Here are some sites I recommend using”) (Johnson and Bylund 2023).

### Study aims

The primary aim of this study was to explore and validate this stoplight typology in the context of adult child caregivers taking the role of a surrogate seeker in a clinical visit with their parent’s blood cancer clinician. We first aimed to establish associations between the typology and caregiver outcome measures we examined the association between the presence or absence of stoplight typology in clinician responses with caregivers self-reported eHealth literacy, perceived communication skills, and quality of physician–caregiver/patient communication. We first aimed to examine the association between the stoplight typology of clinician responses with the caregivers self-reported eHealth literacy, perceived communication skills, and quality of physician–caregiver/patient communication. Our second aim was to describe blood cancer caregivers’ experiences talking with their parent’s clinician about online cancer information. Specifically, we aimed to examine if they have brought online information into the clinical encounter and reasons why they did not, clinician responses to that information, and subsequent information seeking behaviors after the clinician response.

## Methods

### Participants and recruitment

We conducted an online survey between March and June 2020 with adults who were caring for a parent with a blood cancer. Inclusion criteria were (a) caregivers needed to be 18 years or older caring for a living parent, stepparent, or parent-in law with a blood cancer; (b) the parent, stepparent, or parent-in law had to be diagnosed at least 3 months before the start date of the survey; and (c) the parent must have been currently in treatment or completed treatment within the past year. Participants were recruited through Blood Cancer United’s (formerly The Leukemia & Lymphoma Society’s) constituent database via email. Participants who met eligibility requirements received a $25 gift card upon completion of the survey.

### Survey instrument and measures

The survey included items about the participants’ experiences with their parent, stepparent, or parent-in law, especially involving aspects of the communication with their parents’ clinicians. Demographic characteristics of both the caregiver and their relative receiving care were also collected. For this paper, we analyzed responses to the following 3 caregiver-focused measures:

1. The Transactional eHealth Literacy Instrument (TeHLI): a multidimensional measure designed to evaluate skills related to reading, interacting, evaluating, applying, and discussing online health information (Paige et al., 2019). We recently found that a 5-dimension version of the TeHLI (TeHLI-C), used in this study, was valid and reliable in a blood cancer caregiver population (Vasquez et al., 2022b). The TeHLI-C consisted of 23 items evaluated on a 5-point Likert-type scale (1 = strongly disagree to 5 = strongly agree) evaluating 5 dimensions, Functional, Communicative, Critical, Translational, and Clinical. Higher scores indicate higher health literacy.
2. The Patient Report of Communication Behaviors (PRCB): an 11-item measure of patients’ communication in a clinical visit with ratings on a 5-point scale (1 = never to 5 = nearly always). We slightly modified the language of the items for use with a caregiver population (Campbell‐Salome et al., 2021) and added four additional items that represent the caregiver experience. Higher scores indicate a more positive rating of caregivers’ reported clinical communication skills.
3. The Questionnaire of Quality of Physician-Patient Interactions (QQPI): a 12 -item measure using a 5-point Likert type scale (1 = strongly disagree to 5 = strongly agree) that measures the patient or caregiver’s perception of the physician’s communication. Higher scores indicate a more positive rating of the physician’s communication in clinical interactions (Campbell‐Salome et al., 2021).

Participants also responded to open-ended survey questions about discussions of online health information with their parent’s clinicians. For this analysis, we focused on the following questions:

- *Think of the most recent time you searched for information or advice about your parent’s blood cancer on the Internet or read information or advice from the Internet that someone else sent you. Did you speak with a doctor, nurse, or other healthcare provider about this information or advice?* (If no) *Why not?*
- *Have you ever spoken with a doctor, nurse, or other healthcare provider about information or advice you have seen on the internet about your parent’s blood cancer?* (If yes) *How did the provider respond? Please provide as much detail as possible.*
- *Have you ever had a doctor, nurse, or other healthcare provider tell you not to look on the internet for advice or information about your parent’s cancer? (If yes) Did you still look on the internet?*

### Data analysis

First, caregiver responses to the question about clinicians’ responses to online health information (the second one in the list above) were coded based on the stoplight typology (Johnson and Bylund 2023; Bylund et al., 2024) by 2 members of the research team. Intercoder reliability was acceptable (Krippenorff’s alpha = 0.73). A red light response indicates an unwilling to discuss response in which clinicians advise avoiding online health information. A yellow light response signals a cautious response in which clinicians warn about the accuracy of online health information and give reminders to set boundaries and limit the consumption of online health information. A green light response signals a willingness to discuss response in which clinicians will praise research efforts, provide additional online health information sources, or demonstrate that they are taking the online health information seriously.

The TeHLI-C, PRCB, and QQPI were scored. Regression analyses were used to examine the relationship between TeHLI-C, PRCB, and QQPI scores, which reflect participant online health information-seeking behavior, and the stoplight typology of clinician communication behaviors.

Second, we examined participants’ experiences with online health information as reported in open-ended questions. We coded participant responses to the open-ended question about why participants did not discuss online health information with their clinician into 8 categories based on a previously developed coding scheme by 2 members of the research team along with adding new categories (Imes et al., 2008; Bylund et al., 2009). Intercoder reliability was acceptable (Krippenorff’s alpha = 0.80).

## Results

### Survey respondent demographics

A total of 121 participants completed the survey. Participants primarily identified as White, not Hispanic (71%, 92%) and were 44.37 years old on average (SD = 11.48). Twenty five percent of participants reported providing all of the care for the diagnosed parent, 33% providing most of the care for the parent, and 33% providing some care for the parent. Most participants reported that they always attended (38.8%) or attended most (25.6%) of the parent’s healthcare visits. Participants primarily cared for those with leukemia (*n* = 49; 40.50%), myeloma (*n* = 29; 24.0%), or lymphoma (*n* = 28; 23.10%). See Table 1 for full participant demographics.

**Table 1.** Demographic variables

Variable	Total
Age, M (SD)	44.37 (11.48)
**Caregiver sex, *n* (%)**	
Male	21 (18.9)
Female	88 (79.30)
Other	2 (1.80)
Missing	10 (8.30)
**Race, *n* (%)**	
Caucasian	87 (71.9)
Black/African American	11 (9.1)
Asian	11 (9.1)
American Indian	3 (2.5)
Native Hawaiian/Pacific Islander	2 (1.7)
Missing	12 (9.9)
**Ethnicity, *n* (%)**	
Hispanic	19 (15.70)
Not Hispanic	92 (76.00)
Missing	10 (8.30)
**Education, *n* (%)**	
High school graduate/GED	8 (6.6)
Some college	12 (9.9)
2-year degree	9 (7.4)
4-year degree	38 (31.4)
Master’s degree	34 (28.1)
Professional degree	4 (3.3)
Doctoral degree	6 (5.0)
Missing	10 (8.3)
**Employment status, *n* (%)**	
Employed full-time	73 (60.30)
Employed part-time	8 (6.60)
Self-employed	6 (5.0)
Not employed	10 (8.30)
Retired	10 (8.3)
Missing	10 (8.3)
**Amount of care provided to patient, *n* (%)**	
A little	8 (6.60)
Some	40 (33.10)
Most	40 (33.10)
All	33 (27.3)
**Frequency of attending healthcare visits with patients, *n* (%)**	
Never	5 (4.10)
Sometimes	21 (17.4)
About half the time	13 (10.70)
Most of the time	31 (25.60)
Always	47 (38.80)
Missing	4 (3.30)

As part of our first aim, we examined the relationship between the self-report measures of eHealth Literacy (TeHLI) and the participants’ report of clinicians’ use of the stoplight typology responses. Lower eHealth Literacy scores (M = 4.17; SD = .69) were a significant predictor of increased red light responses by clinicians (M = .20; SD = .40), (β = −.32, *t* = 23.186, *p* = .002.). eHealth literacy scores did not significantly predict yellow light or green light response scores.

Also, as part of the first aim, we examined the relationship between a validated self-report measure of perceived clinical communication skills (PRCB) with the spotlight typology of clinician communication. Participants who self-reported better clinical communication skills (M = 4.23; SD = .59) were more likely to report green light responses by clinicians (M = .54; SD = .50), (β = .28, *t* = 2.772, *p* = .007). Reporting lower ratings of clinical communication skills (M = 4.23; SD = .59) was a significant predictor of the likelihood of red light responses (M = .20; SD = .40), (β = −.25, *t* = –2.403, *p* = .018). Self-reported ratings of clinical communication skills did not significantly predict cautionary “yellow light” response scores.

Lastly, as part of the first aim, we examined the relationship between a self-report measure of clinical communication (QQPI) and the spotlight typology of clinician communication. Participant reports of better clinician communication scores (M = 3.94; SD = .50) were significantly associated with a higher likelihood of a green light response by clinicians (M = .54; SD = .50), β = .26, *t* = 2.485, *p* = .015. Moreover, lower clinician communication scores (M = 3.94; SD = .50) were associated with a higher likelihood of a red light response by clinicians (M = .20; SD = .40), β = − .23, *t* = –2.225, *p* = .02. Clinician communication scores did not significantly predict cautionary yellow light response scores.

Our second aim was to further describe surrogate seekers’ experiences talking with their parents’ clinicians about online cancer information. First, we found that 39 of 121 participants (32%) did not speak with their parent’s clinician about the most recent time they searched online for health information. Of those participants who did not talk with a clinician about the information they found online, the most common response was that they felt there was no reason to speak with a clinician about the online health information. See Table 2 for full list.

**Table 2.** Why caregivers did not speak with their clinician about online health information

Code	Definition	Total, *n* (%)
No need	Participant didn’t need to speak to a physician regarding the information they searched for online. Certain reasons this could be coded include: No need, curiosity of the patient/caregiver, didn’t feel like they had to speak to a physician, not necessary.	14 (35.90)
Miscellaneous	Participants’ answer could not be coded.	8 (20.51)
No appointment	Participant did not have an appointment with the clinician to discuss the information. This could be for a variety of reasons: the appointment was still in the future, treatment was still in the future, or the clinician was in another state (in the case of a caregiver).	6 (15.38)
Face saving	Participant wanted to maintain respect of the provider or avoid humiliation. Examples of this could include: feeling embarrassed for seeking information online, not wanting to disrespect the provider, felt discussing online information would insult the provider’s knowledge.	6 (15.38)
Confirmatory	Participant was searching online for information to confirm what was said in a previous appointment with a provider. This could include: Comparison of what a provider said, confirmation that what was said during an appointment aligns with information online, verifying information given is similar for other patients with similar diagnoses.	2 (5.13)
Self-education	Participant was looking up information to inform themselves. This could include: looking for greater understanding of what the physician told the participant, want to have the most information possible about a diagnosis, researching but not seeking guidance from a provider, staying up-to-date with current information, or searching for additional information.	2 (5.13)
For someone else	Participant was not looking up health information online for themselves or their personal use but for someone else. This could be the caregiver looking up information for the patient, participant looking up information for a friend or family member, etc.	1 (2.56)

Ninety of 121 participants (74%) indicated that they had spoken with a clinician about online health information related to their parent’s blood cancer. Of those participants, most reported a clinician response that was coded as a green light response (56%), with 20% as red light responses (*n* = 18), and 23% as yellow light (*n* = 21). See Table 3 for stoplight coding results.

**Table 3.** Clinician approaches as coded with the stoplight typology

	Total, *n* (%)
Red light	18 (20.00)
Yellow light	21 (23.33)
Green light	51 (56.67)
Total answers	90

Finally, we examined what participants do after being told by clinicians not to search for health information online (red light response). Of the 26 participants who indicated a clinician had told them not to look online for information about their parent’s blood cancer, 20 (77%) indicated they still searched online for information, despite the clinician’s guidance.

About 15% of caregivers indicated that they did not have an appointment to discuss the online health information, while another 15% did not discuss the online health information to maintain the respect of the clinician or avoid humiliation (i.e., “save face”).

## Discussion

This study sought to better understand blood cancer caregivers’ experiences when caring for a parent with blood cancer, specifically their experiences around online information seeking as a “surrogate seeker” for the patient. We also described participants’ reported experiences talking with clinicians about online information. We provide support and validation for the stoplight typology of doctor responses to patients going online for information by examining factors that were associated with getting the “green light,” “yellow light,” or “red light” response from clinicians about seeking information online/when they shared online cancer information, as well as self-reported subsequent seeking behaviors after the conversation.

Findings showed that caregivers who reported higher levels of eHealth Literacy perceived clinical communication skills, and communication skills of the clinician were more likely to be willing to discuss online information with the caregiver. Caregivers with self-reported lower levels of eHealth Literacy, perceived communication skills, and communication skills of the clinician were associated with being given the “red light.” Our speculation is that clinicians perceive the lower eHealth Literacy through the participants’ communication and then adjust their recommendations accordingly (Carrard et al., 2018). Moreover, clinicians may be reacting to the poorer communication skills of the participants by discouraging them from going online to read more information.

However, a striking but not surprising finding is that among the caregivers who reported that a clinician had told them not to look online for information about their parent’s blood cancer (a “red light”), most indicated they still searched online for information anyway, despite the clinician telling them not to. This finding is consistent with a previous finding among a population of caregivers and patients of heterogenous cancers that 93% of caregivers and 95% of patients went online anyway after having a red light conversation with a clinician (Bylund et al., 2024). It is clear from these findings that guidance to caregivers not to go online to educate themselves about their parent’s cancer is not an effective communication strategy. Instead, green light responses, or yellow light responses in combination with green light responses, may promote better communication among all parties involved in the patient’s clinical management (Johnson and Bylund 2023; Bylund et al., 2024).

Clinicians who gave a red light response were associated with lower perceived clinician communication skills by the caregiver. Future work could inform clinicians that the red light response is unsuccessful to improve communication about online health information with patients and their caregivers. Other future studies may want to examine specifically those caregivers who reported lower eHealth literacy, seeing as though they most frequently encountered a red light response from a clinician, and may need higher levels of assistance with interpreting and seeking trustworthy, accurate online information about their disease and treatment. Future clinician-focused educational interventions should focus on how to ask about and communicate about online information among low eHealth literate populations, and those with perceived communication skills to ensure that patients understand their disease and make treatment choices based on accurate information.

More than two-thirds of participants spoke with their clinician about online health information, which is consistent with our previous study where 85% of a population of cancer patients and primary caregivers were very or somewhat comfortable discussing online health information with their doctors (Bylund et al., 2009). Yet notably, about one-third of participants did not speak with their clinician about online health information. Although the most reported reason was that participants did not feel a need to discuss online health information, some still reported a barrier to discussing online health information of face saving – meaning they were concerned how the provider would react. Helping providers respond appropriately to patients and caregivers raising online health information can mitigate this barrier. Caregivers need support in identifying trusted information sources and interpreting what they are reading online. This can help caregivers tease out what are trusted sources for information about disease and treatment that are relevant, accurate, up to date, encourage shared decision about novel therapies, and facilitate use of supportive resources and services, versus misinformation.

The present study does have a few notable limitations. All data were self-reported by a relatively small sample of participants. Future studies should examine physician self-reports and conversations with providers and caregivers to gain perspective. All participants were adult children caring for a parent with blood cancer, and the sample was predominantly women and White, limiting the generalizability of the study to other cultures. Future work could expand outside of blood cancer and cancer contexts to determine if the stoplight typology is valid in other health contexts and populations and could include different types of caregivers (e.g., spouse), clinical encounters (i.e., dyadic versus triadic interactions), cancers, or other diseases (i.e., dementia).
